# Extracellular Vesicles and Cancer Therapy: Insights into the Role of Oxidative Stress

**DOI:** 10.3390/antiox11061194

**Published:** 2022-06-17

**Authors:** Jenni Ho, Luksana Chaiswing, Daret K. St. Clair

**Affiliations:** 1 Department of Toxicology and Cancer Biology, University of Kentucky, Lexington, KY 40536, USA; jenni.ho@uky.edu (J.H.); l.chaiswing@uky.edu (L.C.); 2 Markey Cancer Center, University of Kentucky, Lexington, KY 40536, USA

**Keywords:** extracellular vesicles, oxidative stress, 4-hydroxy-2-nonenal, cancer, cancer therapy

## Abstract

Oxidative stress plays a significant role in cancer development and cancer therapy, and is a major contributor to normal tissue injury. The unique characteristics of extracellular vesicles (EVs) have made them potentially useful as a diagnostic tool in that their molecular content indicates their cell of origin and their lipid membrane protects the content from enzymatic degradation. In addition to their possible use as a diagnostic tool, their role in how normal and diseased cells communicate is of high research interest. The most exciting area is the association of EVs, oxidative stress, and pathogenesis of numerous diseases. However, the relationship between oxidative stress and oxidative modifications of EVs is still unclear, which limits full understanding of the clinical potential of EVs. Here, we discuss how EVs, oxidative stress, and cancer therapy relate to one another; how oxidative stress can contribute to the generation of EVs; and how EVs’ contents reveal the presence of oxidative stress. We also point out the potential promise and limitations of using oxidatively modified EVs as biomarkers of cancer and tissue injury with a focus on pediatric oncology patients.

## 1. Introduction

Extracellular vesicles (EVs) are membrane-enclosed particles that contain molecular content that is excreted from most cells and they can modulate downstream targets following uptake [1,2]. Isolated molecular content within EVs may provide insight into the interior state of a cell [1]. The lipid membrane of EVs protects the molecular content from enzymatic degradation, making these organelles promising potential diagnostic and drug delivery tools [3,4]. The role of EVs in normal and pathophysiological cell interactions is being extensively researched [5,6,7,8,9]. For example, while EVs have been identified as playing a role in normal cell-to-cell communication, when homeostasis is altered in a system (such as, an increase in oxidative stress), EV content may change and alter molecular patterns following interactions with downstream targets [2,8,10]. The role of EVs in normal and pathophysiological processes warrants continued research to determine the potential utilization of EVs in translational research, since EVs can be isolated from body fluids, providing biochemical insights into the patients. However, the role of EVs and the molecular content of EVs following cancer therapy have not been fully elucidated, in particular, those EVs with increased levels of oxidative damage following cancer therapy.

Advancements in diagnostic methods, screening technology, and cancer treatments have led to a steady decline in cancer death rate since the 1990s [11]. Specifically, a 2018 review of pediatric oncology patients showed that the death rates for both children (ages 0 to 14) and adolescents (15 to 19) had declined by more than half since 1975 (from 4.9 to 2.0 in children and from 5.9 to 2.9 in adolescents) [11]. Ironically, as cancer therapy becomes more effective, more patients are surviving cancer and living longer, but they are often living with one or more of the unintended consequences caused by therapy. A prominent consequence observed in pediatric cancer survivors is cancer therapy-induced cognitive impairment [12]. While the underlying pathogenesis of cognitive impairment is complex, a growing body of evidence implicates oxidative damage, which results from an imbalance in the reduction–oxidation (redox) regulatory system where the amount of oxidants exceeds the capacity of the antioxidant system to remove the excessive amount of reactive oxygen species (ROS), an important mechanism contributing to damage in the brain microenvironment [13]. In particular, the role of 4-hydroxy-2-nonenal (HNE, a highly reactive end-product of lipid peroxidation) in neurodegenerative disorders is well-established and HNE has been shown to be elevated in a number of different diseases [14,15,16,17,18].

The problem of the consequences of cancer therapy on pediatric and adolescent patients, who are still developing neurocognitive capabilities and who have the potential to live long and productive lives, is critically important to solve [19,20]. It has been demonstrated that oxidative stress may be a contributing factor to the off-target tissue effects in patients receiving cancer therapy, since at least 50% of current chemotherapies are associated with increased ROS production, and radiation therapy utilizes free radicals to exert its therapeutic effects [21,22]. In addition to the direct impact of oxidative stress in contributing to off-target tissue side effects (particularly, to the brain [23,24]), pro-inflammatory cytokines such as TNF-α have also been demonstrated to contribute to the negative consequences of oxidative stress by increasing ROS production and mitochondrial dysfunction [25,26,27].

Current methods to detect alterations in the brain are not sensitive enough to detect these changes early enough to effectively treat or mitigate cancer therapy-induced cognitive impairment, making earlier detection of cancer therapy-induced cognitive impairment vital [28]. Our group is interested in investigating the role of oxidative stress in EV genesis and the potential use of EVs as an indicator of oxidative damage following cancer therapy, which may contribute to the decline in cognitive impairment observed in cancer survivors.

In this review, we briefly summarize the current understanding of the biogenesis, classification, and methodologies used to study EVs; the importance of oxidative stress and how it may contribute to EV generation; and, finally, how EV content relates to oxidative stress and cancer therapy. In particular, we focus on the utilization of oxidatively modified EVs as biomarkers of cancer and tissue injury in children with acute lymphoblastic leukemia.

## 2. Extracellular Vesicles: Biogenesis and Characterization

EVs are membrane-enclosed particles that contain molecular components specific to their cell of origin and circulate freely throughout the body [29]. Originally believed to be a mechanism for the cell to dispose of unwanted molecular products that could not be degraded by other methods, EVs are now known to be conduits of cell-to-cell communication. Nucleic acids (e.g., mRNA and miRNA) are often among their contents, and they have the ability to alter the phenotype of their target cell following internalization [30]. The classification of EVs encompasses a highly heterogeneous group of extracellular particles. The two major groups of EVs are based primarily on their size but also on their biogenesis, contents, and the physiological roles that they play. Even though it remains a challenge to separate the two distinct groups during application [31], it is important to highlight the two main groups that form the generic term “extracellular vesicles”.

Exosomes constitute the first category of EVs. They typically range from 30 to 150 nm in diameter and are derived from the endosomal membrane budding inward, forming intraluminal vesicles (ILVs) during multivesicular endosome (MVE) maturation [1]. These liposomal particles are then secreted from the cell upon the fusion of MVE with the cell surface lipid bilayer. Due to their MVE origin (also known as multivesicular bodies, MVBs), they contain markers that are associated with the endosomal pathway [2]. Exosomes are secreted by various cell types in the body and may play a role in cell-to-cell communication and elimination of undesirable products within the cell, and are part of the normal and pathophysiological processes in the body [2].

**Figure 1:**
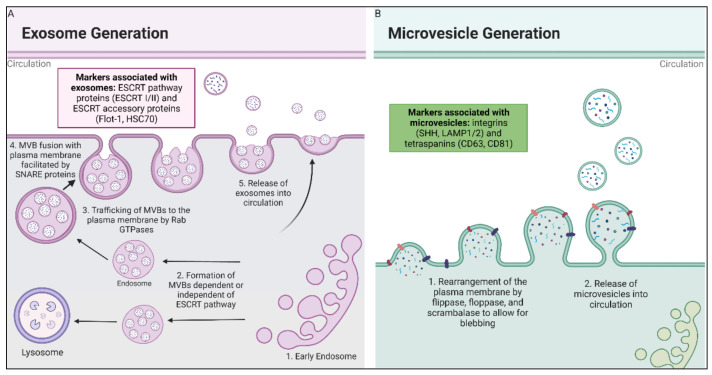
Biogenesis of exosomes and microvesicles. (**A**) Exosomes are derived from fusion of multivesicular endosomal bodies with the plasma membrane, which releases exosomes into extracellular space. Early endosomes are formed by the inward budding of either the plasma membrane or the Golgi Apparatus, where they then mature to late endosomes and become MVBs. The MVBs are then trafficked to the cell membrane, where they are fused with the plasma membrane by SNARE proteins [29,32,33]. (**B**) Microvesicles are formed by the rearrangement of the plasma membrane facilitated by flippase, floppase, scrambalase, and calpain, leading to the budding of the microvesicles from the cell membrane [34].

Microvesicles are the second category of EVs. These molecules are larger in diameter (100 to 1000 nm) than exosomes and have a different route of biogenesis. Microvesicles are formed by budding of the plasma membrane and may possess markers such as integrins and P-selectin [1,2]. The outward budding of the plasma membrane due to apoptosis that creates an apoptotic body has long been known (therefore, is not further addressed in this review); however, the study of microvesicles budding from healthy cells is a more recent area of interest. The current understanding of microvesicle biogenesis is that flippases, floppases, scramblases, and calpain rearrange the composition of the phospholipid bilayer, allowing for physical bending of the membrane, and also allowing for microvesicle formation to occur more efficiently [1]. Figure 1 provides a schematic of the differences in biogenesis between the two different types of EVs.

### 2.1. Biogenesis of EVs

#### 2.1.1. Exosome Biogenesis

The biogenesis of exosomes and microvesicles occurs by two very distinct pathways. Exosomes are derived from ILVs present in MVBs that are formed during the transition of early endosomes to late endosomes. These ILVs are released when MVBs fuse with the plasma membrane and release the ILVs as exosomes into the extracellular space. Because of their origin as ILVs, exosomes have molecular markers related to the endosomal pathway [2]. ILVs form into MVBs by two distinct pathways: one that is dependent on the endosomal sorting complex required for transport (ESCRT) and one that is independent of the ESCRT.

The ESCRT-dependent pathway consists of four complexes (ESCRT-0, ESCRT-I, ESCRT-II, and ESCRT-III) and some associated proteins that sort molecular content into ILVs. ESCRT-0 and its accessory proteins recruit content in a ubiquitin-dependent manner, which begins the formation of ILVs by recruitment of the coat protein, clathrin [35]. ESCRT-I and ESCRT-II cause bud formation, with ESCRT-III driving vesicle scission. Associated proteins (primarily VPS4, an ATPase) dissociate and recycle ESCRT machinery from ILVs [36]. Previous studies that inhibited components of the ESCRT machinery showed that MVBs still formed in mammalian cells, resulting in further exploration of a secondary ILVs formation pathway independent of the ESCRT machinery [37].

In comparison to the relatively well-characterized ESCRT-dependent pathway, how exosomes form independent of the ESCRT pathway is only speculation. Attempts have been made to identify the proteins or lipid molecules that promote inward budding of the MVB membrane and produce ILVs. One such protein is ceramide, which was shown to promote the inward curvature of the MVBs membranes that form ILVs in oligodendrocytes [36]. In the same cells, it was shown that drug-induced or genetic mutation-induced cholesterol accumulation in MVBs resulted in an increase in exosome formation in a flotillin-2 dependent manner [38]. While these are just two possible alternative mechanisms of exosome biogenesis, Kowal et al. outlined other possibilities in their review [36]. It is clear that the formation and release of ILVs in MVBs is a multifaceted process that is dependent on many different factors, which makes it challenging to simply categorize their formation into just ESCRT-dependent and -independent pathways.

Following the formation of MVBs by either pathway, the next step for release of the exosomes into circulation is intracellular trafficking of MVBs to the plasma membrane [29], which is facilitated by cytoskeleton filaments but also is regulated by different proteins [29,39]. The most well-established proteins associated with regulating the trafficking of these intracellular organelles are Rab GTPases, which facilitate vesicle budding, uncoating, motility and fusion [40]. The final step in the release of exosomes into circulation is fusion of the MVBs with the plasma membrane, which is driven by SNARE (soluble NSF attachment protein receptor) proteins [29,32,33].

In summary, exosome biogenesis is derived from the endosomal pathway, leading to exosomes differing from microvesicles by having more molecular markers characteristic of the endosomal pathway that produced them. Following the formation of these MVBs, these organelles are then trafficked to the plasma membrane by cytoskeleton filaments and proteins, where, facilitated by SNARE proteins, they fuse with the cell’s plasma membrane and the exosomes are released into circulation. Whether the ILVs within a single MVB are all from one pathway or a combination of both ESCRT-dependent and ESCRT-independent pathways remains to be addressed by further research.

#### 2.1.2. Microvesicle Biogenesis

While exosomes are derived from ILVs, microvesicles are a result of direct budding of the plasma membrane of the cell; their biogenesis is not as well understood as the biogenesis of exosomes [29]. The first step in the process of microvesicle biogenesis is rearrangement of various plasma membrane components, including lipids and proteins, and changes in calcium levels, which allow for calcium-dependent enzymatic machinery such as flippases, floppases, scramblases, and calpain to alter the composition of the bilayer [6]. Flippases and floppases are aminophospholipid translocases whose main role is to transport phosphatidylserine and phosphatidylethanolamine from one side of the lipid bilayer to the other [1]. Flippases catalyze the movement of these different phospholipid species from the outer leaflet to the cytosolic leaflet, and floppases catalyze the movement in the opposite direction [41]. Both of these enzymes are ATP-dependent and can only transport in one direction. In contrast, scramblases are ATP-independent and can transfer phospholipids bidirectionally. Calpain is a calcium-activated protease which cleaves actin-capping proteins, causing the disruption of the cytoskeleton protein network that leads to membrane budding [34]. The result of these enzymes working together is that the composition of the phospholipid bilayer is rearranged and the actin cytoskeleton is restructured, allowing for the formation of microvesicles budding off the membrane. While the enzymes that help promote formation of microvesicles have been studied, the underlying mechanism and key regulators in the formation of microvesicles is not as well-understood as the biogenesis of exosomes is.

### 2.2. EVs Content

Due to different routes of biogenesis, some molecular content of exosomes and microvesicles may differ, which results in some differences in their membrane contents. However, these two different forms of EVs also share similarities in molecular content. Overall, both forms contain proteins, lipids, and nucleic acids characteristic of their cell of origin, but they do have distinct profiles based on their biogenesis.

The composition of the contents of the lipid membrane of EVs shares many similarities with their cell of origin. Exosomes typically contain sphingomyelin, gangliosides, and disaturated lipids; however, they have lower levels of phosphatidylcholine and diacylglycerol compared to their cell of origin [42]. Indicative of their route of biogenesis in comparison to exosomes, the lipid composition of microvesicles shows more similarities with the cellular membrane from which the microvesicles are derived. They do, however, have increased polyunsaturated glycerophosphoserine and phosphatidylserine, as these lipids are more prominent in lipid rafts and are not evenly distributed in the lipid membrane of the cell [43]. Despite the two different biogenesis routes for exosomes and microvesicles, overall the phospholipid components of exosomes and microvesicles remain relatively constant [34].

In contrast to the similar phospholipid composition of EVs, the proteins in these liposomal particles are more heterogeneous. For example, exosomes contain proteins that are associated with the ESCRT pathway (such as TSG101 and ALIX), while microvesicles are associated with integrins and selectins [2]. However, microvesicles and exosomes do have some similarity in their protein content due to their formation. Both categories of EVs contain different kinds of tetraspanins (CD63, CD81, CD9), the antigen-presenting proteins involved in signal transduction (e.g., EGFR), and other transmembrane proteins, such as LAMP1 and TfR [44]. Along with the proteins associated with their biogenesis, EVs contain proteins specific to their parental cell. For example, EVs released from antigen-presenting cells contain membranes enriched in MHC-I, MHC-II, and co-stimulatory molecules, while those released from tumor cells contain pro-apoptotic molecules, such TNF-related apoptosis-inducing ligand (TRAIL) and FasL in microvesicles derived from colorectal cancer cells [34,45].

EVs are also enriched with small RNAs such as mRNA, microRNA (miRNA), rRNA, and non-coding RNA (ncRNA), which are typically fragmented, but not with DNA [44]. Similar to proteins, the nucleic acid content of EVs reflects the types and levels of nucleic acid in the cytoplasm of the parental cell and can provide insight into the physiological state of the cell of origin; however, some studies have illustrated that the RNA content of EVs does differ somewhat from that of the parental cell [5,44,46]. In their findings, Pigati et al. demonstrated that EVs released by normal epithelial cells did not contain elevated levels of miR-451 (shown to downregulate macrophage migration inhibitory factor, MIF, and multi-drug resistance, MDR1, in cancer cells), but were released in malignant mammary cells [46]. This finding demonstrates how different cell types may selectively export nucleic acids via EVs, releasing nucleic acid that may help promote cancer growth whereas their normal cell counterparts do not release these microRNAs.

### 2.3. EVs Characterization

The International Society of Extracellular Vesicles (ISEV) published the Minimal Information for Studies of Extracellular Vesicles (MISEV) guidelines in 2014, and updated them in 2018 [31]. Based on the different components of EVs, Thery et al. proposed the standard measurements and quantifications needed to characterize EVs to promote rigor and reproducibility. They discussed the challenge of isolating one class of EVs due to the limitations in isolation methods and techniques that make it extremely difficult to ensure that a population of EVs is exclusively exosomes or microvesicles. However, the differences in biogenesis and origin of these two categories enable measurement of certain characteristics of EVs that may provide some insight into which category of EVs may be more prominent in a sample. Table 1 shows the MISEV2018 characteristics that determine the purity and efficacy of EV isolation and the suggestions of the society for any publications that report studies of EVs.

When studying EVs, it is first necessary to quantify the amount of EVs isolated. MISEV2018 emphasizes that such methods as quantifying lipid content, DNA content, or specific markers of EVs unavoidably lead to contamination [28]. Therefore, a more accurate way of quantifying the number of EVs is by the protein concentration (such as with a BCA or Bradford assay) and the particle number (e.g., nanoparticle tracking analysis). Our group uses the BCA method to quantify EVs’ protein concentration to complement findings from our nanoparticle tracking analysis method that uses Zeta View by Particle Metrix. Moreover, protein concentration normalized by the particle number may also provide insight into how much of the protein detected is part of the population of isolated EVs and is not contaminated starting material.

The second step in the study of EVs is to investigate the different protein markers associated with EVs, and a method such as Western blotting can be used to quantify protein expression in an isolated sample. Measuring these markers in a given population of isolated EVs can be challenging, as the quantity of isolated EVs can be limited. Improvements in Western blot techniques increase sensitivity to these markers from a small sample size, and have been achieved by using Jess by Protein Simple [51]. Utilizing this system has allowed for effective quantification of proteins of interest, even when EVs were isolated from a limited amount of mice sera, highlighting the effectiveness of the system compared to a traditional Western blot [47]. Exosomes and microvesicles have different positive markers that may indicate which population is being analyzed (summarized in Table 1). Markers indicating exosomes include cytosolic proteins, while markers of microvesicles include transmembrane or lipid-bound proteins. The use of at least three positive markers of EVs is recommended and, furthermore, at least one should be a cytosolic protein and one a transmembrane protein. Finally, the use of a negative or contamination protein marker is also recommended in order to indicate how much contamination is present in the isolated sample. In summary, at least three positive markers and one negative (contamination) marker are required for studying the protein composition of EVs. In addition, MISEV2018 recommends two other categories of markers that are not required but would provide optimal conditions for functional studies of EVs. The first category encompasses proteins localized in or on intracellular compartments to further demonstrate the population is predominantly exosomes and derived from MVBs; the second is secreted or luminal proteins on the EVs surface for mode of association of EVs [31].

The first two steps outlined above (quantification of EVs by measuring EVs’ protein concentration and measurement of purification by quantifying EVs markers) assess the purity of the sample by looking at the entire population of isolated EVs. The third step aims to provide details about individual EVs that are present in this bulk population. It is suggested that at least two different but complementary methods can be utilized to characterize individual vesicles. The first method is utilization of a technique such as scanning electron microscopy, transmission electron microscopy, or cryo-electron microscopy to provide a high-resolution image of individual vesicles. Additionally, a technique called atomic force microscopy (AFM) has recently shown promise to visualize the topography of EVs, highlighting its use in analyzing single EV molecules [50]. Utilization of one of these methods allows for analysis of the morphology of EVs that were isolated. The second method uses a complementary assay that measures individual biophysical properties of the EVs. For example, nanoparticle tracking analysis utilizes the light scattering properties and Brownian motion of the EVs to measure the size distribution and particle number in a given liquid suspension; these properties are measured in each individual vesicle in order to determine information about the whole population of isolated EVs.

The final information required for the study of EVs, as suggested by MISEV2018, is characterization of the topology of EV-associated components. This serves to provide further evidence of the functionality of EVs and the proteins encompassed within them. For example, to establish the location of the proteins in the EVs, Cvjetkovic et al. used EVs isolated from HMC-1 mast cells through differential ultracentrifugation, followed by one of the following treatments: (1) no treatment of the isolated EVs; (2) EVs treated with proteinase K (degrading proteins on the surface of EVs); and (3) EVs treated with trypsin/Lys-C (digestion of surface proteins) followed by a biotin tag [52]. Using MS/MS analysis, they found that the proteomic profiles of the treatment groups differed. This finding gave them some insight into which proteins overlapped on the surface of the EVs compared to inside the EVs; it also demonstrated that the location of proteins in the EVs vary, which may be responsible for potential downstream signaling [52]. The protocol followed by Cvjetkovic et al. is one example of how the topology or location of the protein of interest may be vital in understanding the mechanism of EVs. Thery et al. provide other examples of how to measure this topology by using different enzymes and detergents to acquire samples with different surface and internal levels of degradation. For further details on this type of methodology, please refer to MISEV2018 [31].

### 2.4. EVs Isolation

A number of different isolation methods are currently employed to study EVs, and the method used varies depending on: (1) type and quantity of the EVs fluids that were isolated; (2) resource limitations; and (3) study endpoints. Here, we briefly summarize some of the methods utilized for EVs isolation.

The most common method of EVs isolation currently used internationally is ultracentrifugation, with approximately 81% of researchers utilizing this method due to its low cost, purity, and ability to handle a large volume of starting material [53]. Carnino and colleagues demonstrated that while there are a number of benefits, there are still limitations, such as access to ultracentrifugation equipment, tedious step-by-step protocols, and successful extraction of EVs being dependent on the rotor size of the ultracentrifuge and resulting g-force [54]. Based on an ISEV survey, other common methods used are density gradient centrifugation (20% of researchers), filtration (18%), and size exclusion chromatography (15%) [54]. Table 2 summarizes the advantages and disadvantages of these methods, as well as ultracentrifugation and some commercially available kits that utilize polyethylene glycol (PEG) precipitation that are used to decrease the solubility of EVs so they can be isolated by precipitation [53,54,55].

A number of different commercial kits are available through manufacturers, such as System Biosciences, ThermoFisher, HansaBioMed, Exiqon, and Qiagen, to name a few. While the table is not all-inclusive, it summarizes notable advantages and disadvantages of the most common types of isolation methods used by researchers. It is important to note that the majority of the researchers who responded to the questionnaire (all members of ISEV) utilized a combination of isolation techniques (59%) [53]. As previously mentioned, when selecting a method of EVs isolation, a number of different parameters should be taken into account. For example, isolation of EVs from human serum may limit the volume of the starting sample, whereas this is not typically a limiting factor when isolating EVs from cell culture media. Additionally, depending on the downstream applications, a higher and more pure yield of EVs may be required for additional experiments. Nevertheless, it is important to note the variety of different methods available to researchers for EVs isolation, and customized optimization of these different techniques should be employed to identify the best approach for the researcher’s purpose [53,54,55].

In summary, EVs have emerged as a research area of interest because their molecular content identifies their cell of origin and they have potential downstream consequences. The next section of this review will emphasize how the generation and modification of EVs may provide insights into cellular damage, leading to the potential monitoring of disease development, treatment outcome, and tissue damage in a clinical setting.

## 3. Oxidative Stress: Pro-Oxidants and Antioxidants

Oxidative stress results from increased ROS or reactive nitrogen species (RNS), where the ROS or RNS pro-oxidant conditions cannot be mitigated due to inefficient antioxidant systems or dysregulation of the redox pathways that control the balance between pro-oxidants and antioxidants. The resulting oxidative stress can cause oxidative damage, which has been demonstrated to increase with age and has been linked with numerous neurodegenerative diseases [16,17]. Oxidative stress has also been linked with increased risk of developing cancer, which is due, in part, to genetic mutations that favor the development of cancer and which contribute to a pro-tumorigenic microenvironment [57,58,59]. Therefore, the careful regulation of redox balance is critical for normal physiological function.

### 3.1. Oxidants and ROS

Oxidants are molecules that are capable of oxidizing or taking electrons from other atoms or molecules, causing a change in the molecule charge. Many oxidants are generated through normal physiological pathways in the body and are primarily produced in the mitochondria. For example, through the buildup of the electron gradient to generate ATP during oxidative phosphorylation, the electron transport chain produces superoxide anion radical (O_2_^•−^) primarily at complex I (mainly in the matrix) and at complex III (mainly in the matrix and intermembrane space) of the mitochondria [60].

Free radicals are molecules that contain at least one unpaired electron. Examples of free radicals are the hydroxyl radical (^•^OH) and O_2_^•−^. ROS are molecules derived from molecular oxygen (O_2_) but have more reactivity than O_2_ due to the atomic orbital these electrons occupy. Whether ROS are considered free radicals depends on whether the ROS has an unpaired electron (i.e., hydrogen peroxide, H_2_O_2_, does not have an unpaired electron and is therefore a ROS but not a free radical). Hund’s rule states that every electron orbital must be occupied by a single electron before any one orbital is doubly occupied and unpaired electrons contribute to the high reactivity of free radicals and ROS. An increase in either type of molecule beyond its antioxidant capacity can cause damage to macromolecules or reactions with certain chemical groups that activate or inactivate protein functions [61,62,63].

### 3.2. Antioxidants and Redox Couples

Due to the negative consequences of high levels of ROS, the body possesses numerous antioxidant mechanisms to combat oxidants. Here, we summarize some key antioxidant enzymes that are critical for redox regulation and mention some non-enzymatic antioxidants and the six major redox couples present in cells of the human body. The major group of enzymes that detoxify superoxide radicals in the cell is a family of three enzymes known as superoxide dismutase (SODs), which catalyze the conversion of O_2_^•−^ to H_2_O_2_ and O_2_. Copper zinc SOD (SOD1) is a homodimer that has Cu^2+^ and Zn^2+^ at the catalytic site and is located in the cytoplasm, the intermembrane space of the mitochondria, in the nucleus, and in lysosomes [64]. Manganese SOD (SOD2) is a homotetrameric enzyme containing Mn^2+^ and is located within the mitochondrial matrix. Extracellular SOD (SOD3) is a Cu^2+^- and Zn^2+^-containing homotetramer residing on the cell surface [65]. The dismutation of the superoxide radical into H_2_O_2_ is particularly important due to the downstream effects of hydrogen peroxide. H_2_O_2_ is oxidized by ferrous iron to produce ferric iron, a hydroxyl radical, and a hydroxide ion (Fe^2+^ + H_2_O_2_ → Fe^3+^ + ^•^OH + OH^−^). The ferric iron can itself be reduced by O_2_^•−^ to produce ferrous iron and O_2_ (Fe^3+^ + O_2_^•−^ → Fe^2+^ + O_2_). This pair of reactions results in the following net reaction (known as the Fenton reaction): H_2_O_2_ + O_2_^•−^ → ^•^OH + OH^−^ + O_2_ [66]. The Fenton reaction is a seminal contribution to the field of free radical biology and it has been accepted as the primary mechanism of cellular oxidative stress in organisms. Due to the high production of ROS from the mitochondria, the localization of MnSOD in the mitochondrial matrix establishes the importance of MnSOD as the most vital SOD enzyme, and our lab has worked for over two decades to investigate the relationship between MnSOD and its role in cancer [67,68].

Following the dismutation of superoxide to O_2_^•−^ to H_2_O_2_ and O_2_, two H_2_O_2_ molecules are then broken down into two molecules of water and O_2_ (2H_2_O_2_ → 2H_2_O + O_2_) by catalase. Catalase is another vital antioxidant enzyme that is present in almost all aerobic organisms that mitigate oxidative damage. Catalase dysfunction or inhibition has been implicated in aging, another indication of the effects of oxidative damage in human disease [69]. Additionally, H_2_O_2_ can be reduced to two water molecules catalyzed by glutathione peroxidase (GPx), where glutathione (GSH) is used as a cofactor and is oxidized to glutathione disulfide (GSSG) (H_2_O_2_ + 2GSH → 2H_2_O + GSSG) [70]. The oxidized glutathione (GSSG) is then converted back to its reduced form, catalyzed by glutathione reductase, with FAD as a cofactor.

In addition to the SOD enzymes, catalase, and GPx, the body also utilizes non-enzymatic antioxidants such as vitamin C, vitamin E, carotenoids, and lipoic acid to assist in the redox regulation of a system [71]. Moreover, the cell contains six major redox couples that ensure the availability of electrons within the cell: (1) NADH/NAD; (2) NADPH/NADP; (3) cysteine/cystine; (4) GSH/GSSG; (5) peroxiredoxin (Prx)/sulfiredoxin (Srx); and (6) thioredoxin (Trx)/thioredoxin disulfide (TrxSS). These six major redox couples are in different subcellular and extracellular compartments where they minimize some of the potential for damage caused by free radicals. Prevention of oxidative stress and damage to the cell depends on the balance among these different molecules being tightly regulated to promptly remove ROS produced by the cell.

### 3.3. Redox Signaling

Despite the negative consequences of a surplus of ROS, many ROS serve additional positive roles in normal physiological functions [72,73]. For example, at lower levels, H_2_O_2_ plays an important role in a variety of cellular functions, such as cell proliferation, differentiation, migration, and apoptosis [74,75]. However, because homolytic fission of H_2_O_2_ produces two ^•^OH [76], the level of H_2_O_2_, as well as the by-products of H_2_O_2_, must be tightly regulated.

While antioxidants balance the levels of ROS, an abundance of ROS can result in DNA damage when double bonds are added to DNA bases or if a hydrogen atom is abstracted from the DNA [77]. Additionally, elevated levels of ROS have been shown to play a role in tumor development and progression, primarily by acting as a second messenger in signaling pathways that promote cell proliferation and survival by oxidatively modifying regulators of these pathways (e.g., MAPK/ERK, PI3K/AKT, and NF-κB activation pathways) [78]. For example, in the presence of high levels of ROS, the MAPK/ERK pathway has increased activity of Erk1/2 because of continuous ubiquitination and loss of endogenous mitogen-activated protein kinase phosphatase 3 (MKP3), which negatively regulate Erk1/2 activity [79].

Cancer cells have also been demonstrated to have a higher antioxidant capacity compared to normal cells, which overcomes apoptotic signals induced by oxidative stress [80,81]. As a result, monitoring the balance of redox state within a cell may provide significant insight into cancer progression or severity of disease. Overall, the contribution of ROS to cancer progression and development is understood to be facilitated by the induction of DNA mutations, genetic instability, epigenetic changes, and cell proliferation; additionally, stabilization of cells withstands the elevated amount of oxidative stress and allows for cancer survival under stress conditions [82]. While there is a significant amount of literature elucidating the significant role of oxidative stress in cancer biology, to identify potential targets of intervention, much remains to be understood about which players and mechanisms are more susceptible to oxidative damage.

### 3.4. Oxidative Stress and Cancer Therapy

The effect of oxidative stress in cancer therapy is especially critical due to the utilization of radiation therapy as a common cancer treatment modality. Ionizing radiation (IR) effectively causes cancer cell death by directly ionizing macromolecules or generating ROS through the homolytic fission of water into the hydrogen radical (H^•^) and the hydroxyl radical (^•^OH), where ^•^OH is then a potent oxidant and can damage molecules, for instance, by reacting to guanosine sugar in DNA and causing DNA breakage [22].

In addition to radiation therapy, at least 50% of chemotherapies currently used are associated with the generation of ROS [21]. Examples of chemotherapy agents that generate ROS are anthracyclines (e.g., doxorubicin and daunorubicin) and alkylating agents (e.g., cyclophosphasmide, carboplatin, and cisplatin) [83,84]. While the utilization of oxidative stress aims to destroy cancer cells, off-target normal tissue injury may occur as a result of the free radicals generated by these treatment modalities. For example, the generation of O_2_^•−^ by doxorubicin, which removes one electron from the NADH dehydrogenase at complex I and donates it to O_2_, can result in increased ^•^OH because the SOD enzymes catalyze the dismutation of O_2_^•−^ to H_2_O_2_ [85]. As a result, this can initiate an oxidation chain reaction, especially on membrane lipid bilayers, causing LPO that compromises the integrity of the lipid membranes, but also products such as the toxic aldehyde HNE that can lead to further target-specific damage [86]. Generation and accumulation of these toxic aldehyde by-products can have a myriad of downstream effects and may ultimately contribute to oxidative stress-mediated normal tissue injury as a result of cancer treatment, since the accumulation of these by-products (particularly, HNE) has been implicated in neurodegenerative diseases such as Alzheimer’s [14,17].

### 3.5. HNE and EVs

It is well-established that HNE is derived from the oxidation of omega-6 polyunsaturated fatty acids, such as lipids containing linoleic acid and arachidonic acid, which are commonly found in the cell membrane. An increase in HNE and its downstream effects following HNE adduction have been implicated in disease pathologies [87,88,89,90,91]. The high reactivity of this aldehyde molecule is predominantly through two different reactions: it has the ability to covalently adduct to histidine, cysteine, or lysine resides of proteins via a Michael addition due to the double bond between the C2 and C3 carbons; and it can form Schiff bases with the N-termini of peptide chains and the ε-amino groups of lysine residues of proteins [14,92].

Despite being a by-product of LPO, these reactions between HNE and protein residues demonstrate the impact of increased oxidative stress causing protein misfolding and ultimately modifying protein activity. Additionally, HNE accumulation has long been established as inhibiting proteasome function, which results in modifying protein turnover, due to its preventing proper degradation of these protein aggregates [93]. Particularly, HNE has been linked with Alzheimer’s and other neurodegenerative diseases associated with protein aggregation formation [94,95,96,97]. Other examples of diseases involving HNE are atherosclerosis and heart disease [98,99]. A previous study by our group demonstrated that doxorubicin treatment affected HNE-adducted proteins by altering energy metabolism, which ultimately led to the cardiac tissue injury that is seen as a major obstacle when doxorubicin is used as a chemotherapy agent [100]. Moreover, another study by our group demonstrated how an increase in HNE-adducted proteins was found in EVs isolated from mice treated with doxorubicin compared to the control mice [101]. These two studies suggest that not only is HNE a contributor to oxidative stress-mediated tissue injury but that monitoring HNE-adducted proteins levels may provide insight into off-target tissue injury that results from cancer treatment. Overall, high levels of HNE can have a significant impact on disease development and they may also be an indicator of potential downstream negative impact because of increased oxidative stress.

As previously mentioned, one of the consequences of increased HNE is the effect on proteasome function, which could lead to an increase in the number of damaged proteins that accumulate within the cell due to the dysregulation of the cell’s removal processes [97]. When this happens, another method for the cell to dispose of oxidatively damaged molecules is by exportation through EVs. One of the previous studies by our group demonstrated that treatment with the oxidative stress-inducing chemotherapy agent doxorubicin resulted in increased EV generation [101]. This correlation between increased oxidative stress and increased EV generation suggests that EVs may be a method by which cells dispose of oxidatively modified proteins in the presence of proteasome damage induced by increased oxidative damage. However, the consequences of oxidative stress on EVs generation and formation are not fully understood. Studies to elucidate the impact of oxidative stress on EV biogenesis are underway in our lab and the findings from these studies will lead to novel redox-based therapeutic targets to alleviate disease burden [102].

## 4. Role and Function of EVs: Current Understanding and Future Directions

The fundamental role of EVs in both normal and pathophysiological conditions has been demonstrated, and more research is underway to explore the potential use of EVs in the clinical setting. EVs play a variety of roles in maintaining normal physiological functions within the body. Their content allows for delivery of effectors (e.g., transcription factors, nucleic acids, oncogenes) to recipient cells or for the proteins or lipids they contain to activate surface receptors [103]. Many of the earlier studies elucidating the role of EVs in both normal and pathological physiologies emphasized the use of EVs to transport nucleic acids to target cells, which results in alterations in gene expression. Valadi et al. demonstrated the various kinds of nucleic acids (i.e., mRNA, miRNA) found in exosomes released from both human and murine mast cells, and elucidated how delivery of these nucleic acids altered the protein expression in target cells after they were harvested [30]. The role of EVs in the immune system has also piqued interest in recent years; their ability to present antigens and trigger different immune responses is of interest to immunologists, who are investigating their potential use in the clinic [9]. Additionally, miRNAs within exosomes have been demonstrated to modulate inflammation responses, revealing their contribution to compensatory mechanisms that maintain homeostasis [104]. EVs have also been shown to be released from a variety of stem cell populations, where they help maintain plasticity and are able to promote proliferation and vascularization in damaged tissues [105]. Moreover, EVs have also been shown to take part in the coagulation cascade, neuronal communication in the brain, and cell phenotype modulation [8,103].

### 4.1. EVs in Cancer

Along with their function in normal physiological processes, EVs have also been shown to be mediators in a variety of disease pathologies. Numerous studies have demonstrated the role that EVs play in cancer, mostly how they promote tumorigenesis by altering the microenvironment to be more favorable to metastatic growth [6,106]. EVs secreted directly from tumor cells have also been shown to promote cell proliferation, stimulate angiogenesis, promote matrix remodeling, and suppress or modify the immune system to a pro-tumorigenic phenotype [5,6,7,106,107]. Along with the tumor cells releasing EVs, macrophages associated with tumors have been shown to promote tumor invasiveness by delivering oncogenic miRNAs [108]. These findings demonstrate the different pathways and methods by which EVs can be utilized by a tumor to promote its survival. Additionally, EVs have been implicated in other roles, such as activating immune cells in inflammatory diseases and facilitating neurodegenerative diseases by delivering toxic aggregates [109,110].

It is well-established that a number of transcription factors are sensitive to redox alterations (e.g., Nuclear factor kappa B, NF-κB, and Nuclear factor erythroid 2-related factor 2, Nrf2); therefore, monitoring the levels of oxidative stress in cancer cells may provide information about the characteristics of the cancer [102]. In particular, a number of therapy-resistant cancers have been shown to have increased levels of ROS but also an up-regulation in a number of antioxidant enzymes that can avoid oxidative stress-induced apoptosis [80,111]. This dysregulation of redox proteins may facilitate some of the therapeutic resistance seen in aggressive cancers, and, ultimately, may provide insight into an optimal therapeutic approach to overcome this resistance. While a benefit of monitoring oxidative stress levels utilizing EVs would provide an accurate, real-time quantification of the oxidative damage induced by both the cancer and cancer therapies, a limitation is the inability to identify the origin of the EVs. EVs can be isolated from a number of different bodily fluids, with the most common being blood. However, the EVs isolated from patient serum or plasma may have been released from any tissue present within the patient. While identification of tissue-specific markers may allow for some identification of the tissue of origin, the efficacy of detection along with acquiring enough EVs from one specific tissue would be significantly challenging [31]. Additionally, some markers of oxidative stress may be difficult to measure because the kinetics of some of the reactions allow for only a short time frame for detection. As a result, oxidative stress markers must be carefully selected to ensure they are able to reflect the redox status of the system. Nevertheless, despite some of these challenges, the potential utilization of EVs and the oxidative modifications within EVs, such as the HNE-adducted EVs, may provide vital insights into the redox status of the patient as a result of the cancer and cancer treatment, thus emphasizing the importance of continuous studies advancing the use of EVs as a diagnostic tool.

### 4.2. Translational Uses of EVs

Current studies are hoping to elucidate and optimize drug delivery utilizing EVs as a means to directly target certain tissue types or increase therapeutic efficacy. A study by Ohno et al. demonstrated that miRNAs delivered in EVs decreased breast cancer development in a mouse model [112]. Another study by Yao et al. utilized a similar application, where EVs derived from anti-inflammatory M2 macrophages were able to inhibit cell migration and invasion in a glioma cell line by delivery of miRNA targeting the PI3K/AKT/mTOR signaling pathway [113]. While these are just two examples of engineering EVs to be utilized for therapeutic purposes, many research groups are conducting research in the hopes of identifying clinical applications of engineered EVs as a drug delivery tool [4,114,115].

Since the content of EVs is dependent on their cell of origin, characterization of EVs may allow for insights into the state of their cell of origin, such as in monitoring disease progression [116]. Balaj et al. demonstrated that EVs generated from cancer cells in vitro possessed nucleic acid content that reflected the genetic landscape of the tumor, while they also contained genetic information that may be used for horizontal gene transfer and as a potential biomarker [117]. In addition to in vitro studies, it has been demonstrated that EVs isolated from the blood of pancreatic cancer patients can provide insight into the genetic mutations in a patient’s tumor, allowing for precision therapeutic options [118].

Proteomic profiling of EVs has also shown diagnostic potential: Liu et al. demonstrated how a small volume of serum from cancer patients and from healthy individuals (*n* = 102) allowed for accurate detection of cancer (99% accuracy) and was further able to specify the cancer type (68% accuracy) [119]. Previous findings in our lab utilized EVs to detect myocardial damage induced by the chemotherapy drug doxorubicin in mice by detecting glycogen phosphorylase (PYGB) in EVs, where an increase in PYGB in EVs correlated with a decrease in its expression in cardiac tissue compared to control mice [101]. Interestingly, this study demonstrated that EVs were a more sensitive indicator of cardiomyocyte damage following doxorubicin treatment compared to the current method, which considers troponin levels. This study highlights the limitation of using doxorubicin in the clinic—namely, its deleterious effects on cardiac tissue—and suggests that EVs may be useful as an early indicator of these effects, potentially allowing for mitigation of these consequences in patients receiving doxorubicin. These studies illustrate the potential diagnostic use of EVs to monitor off-target tissue damage.

### 4.3. EVs and Cancer Therapy

While nucleic acid, protein, and lipid markers enable the use of EVs as a diagnostic tool in the clinic, it is critical that modifications to these molecular components not be overlooked, as they may provide understanding about other damage that may be occurring in the model from which they were isolated. For example, an increased amount of oxidative stress markers (e.g., HNE-adducted proteins) may indicate that oxidative damage is occurring in the system. Particularly, as some forms of cancer therapy contribute to increased oxidative stress, oxidatively modified EVs can be an excellent tool to assess the redox status and the potential side effects associated with cancer therapy.

Our group recently studied EVs derived from murine models that received a single 10-Gray dose of cranial radiation, a dose that mimics human whole brain radiation and causes neurocognitive alterations in mice [120,121]. Our study illustrated that there were no HNE adduction changes in the brain tissue of the mice (using IHC staining) or glial fibrillary acidic protein (GFAP, a marker of reactive astrocytes; IHC staining and homogenized brain tissue lysate were used for measurement), but when the mice were euthanized 48 h after cranial radiation, EVs isolated from the mice had statistically significant elevated levels of HNE-adducted proteins and GFAP compared to the EVs isolated from the control mice. This study shows: (1) the advantages of utilizing HNE adducts in EVs as a marker of oxidative stress that alters redox homeostasis in the system; and (2) the potential use of EVs as an early indicator of astrocyte reactivity as a result of damage to the brain microenvironment induced by cranial irradiation [47].

As previously mentioned, our group has demonstrated how an increase in HNE adduction mediated cardiac damage following doxorubicin treatment and that an increase in HNE adducts was present in EVs isolated from mice treated with doxorubicin compared to the control mice [100,101]. These findings suggest that: (1) an increase in HNE as a result of increased oxidative stress is a mediator of normal tissue injury during cancer therapy; and (2) quantification of oxidative modifications (e.g., HNE adducts) in EVs has clinical potential as a diagnostic tool to monitor downstream implications of increased oxidative stress and disease progression. Additionally, one of the most common off-target tissue injuries seen is damage to the brain induced by cancer treatment. Therefore, monitoring damage to the brain microenvironment is critical, especially in pediatric cancer.

Acute lymphoblastic leukemia (ALL) is the most commonly diagnosed form of pediatric cancer, with a median age of diagnosis of between 2 and 5 years of age [11]. Overall, the survival rate of pediatric ALL is high (~90%); however, numerous published studies report significant neurobehavioral and neurocognitive differences between these survivors and healthy age-matched counterparts [19,20,28]. Despite a majority of pediatric ALL patients no longer receiving cranial radiation as a proactive treatment to decrease the potential of CNS invasion of the leukemia, the percentage of pediatric ALL survivors that report neurocognitive decline (~33%) emphasizes the necessity for early detection of these off-target tissue effects to allow for intervention [122,123,124,125].

The current treatment regimen for pediatric ALL patients encompasses multiple phases of therapy (induction, consolidation, and maintenance), and several different chemotherapy agents are given to these patients during this time [126,127]. Induction is the first phase of the ALL treatment, with the goal for the patient to be in remission, with a current success rate of 95% [126]. The results from the clinical trial, AALL0932, recommend cytarabine, methotrexate, pegasparagase, vincristine, and dexamethasone during the induction phase [128]. Some protocols may also include the addition of an anthracycline, such as daunorubicin or doxorubicin [127]. Cytarabine has previously been demonstrated to induce apoptosis by increasing mitochondrial ROS production, where the addition of N-acetyl cysteine (NAC) reversed the increase in apoptosis that was previously seen when cells were treated with cytarabine [129]. Daunorubicin has also been shown to contribute to mitochondrial dysfunction, ultimately leading to increased ROS production that induces DNA damage and cell death [84]. Additionally, dependent on the protocol, the consolidation and maintenance phases may introduce more chemotherapy agents, such as mercaptopurine. Altogether, it is clear these patients receive a diverse combination of drugs to kill the leukemia cells. Moreover, following the AALL0932 guidelines, cytarabine and methotrexate are given to patients via an intrathecal injection, leading to direct exposure of these chemotherapy drugs for the central nervous system (CNS). The mechanism of action of a number of these chemotherapy drugs, combined with the direct exposure to the CNS by cytarabine and methotrexate, may contribute to the decline in neurocognition observed in these patients [130,131,132].

The significance of increased ROS production as a product of cancer therapies (e.g., radiation and different chemotherapy agents) may ultimately contribute to the decline in neurocognition that is seen in pediatric ALL survivors. Oxidative stress has been well-established to have a number of downstream consequences to the brain that can ultimately contribute to neuronal decline, such as: (1) inducing apoptosis; (2) proteasome malfunction; (3) mitochondrial dysfunction; (4) protein misfolding; and (5) glial cell activation [23]. In addition to proteasome malfunction, oxidative stress has been demonstrated to lead to lysosome dysfunction as a result of oxidation of the lysosome membranes [133]. Proper lysosomal and autophagy functions have been shown to be critical for proper myelination of neurons by oligodendrocytes and lead to neurodegenerative disease in vivo [134,135]. As previously mentioned in Section 3.5 of this review, the increase in oxidative stress may ultimately lead to the accumulation of HNE within the cell. Our group, therefore, hypothesizes that the accumulation of misfolded proteins by HNE adductions, in combination with proteasome and lysosome dysfunction, leads to the excretion of misfolded proteins in EVs, where isolation of EVs may provide the opportunity to: (1) quantify oxidative stress markers (i.e., HNE-adducted proteins) that would provide insights into the redox dysregulation occurring throughout a patient’s treatment; and (2) increase sensitivity to detect proteins associated with off-target tissue damage (e.g., GFAP indicating astrocyte activation as a result of increased ROS and potential neuronal injury).

Previous studies assessing this hypothesis have thus far demonstrated: (1) the ability of EVs to be a more sensitive indicator of oxidative stress and myocardial death following treatment with the chemotherapy agent doxorubicin in vivo than the current method of measuring troponin levels [101]; and (2) EVs are a more sensitive indicator of astrocyte activation and oxidative stress than the brain tissue and serum of mice euthanized 48 h following cranial radiation [47]. Thus, our group has hypothesized the putative ability of EVs to be used as a sensitive indicator of oxidative stress and off-target tissue damage following treatment with both chemotherapy [65] and radiation [47] utilizing in vivo models. Further studies of EVs derived from pediatric ALL patients are needed to validate the translational use of EVs to detect alterations and may identify earlier targets of intervention to mitigate the by-product of off-target tissue damage in cancer patients.

## 5. Conclusions

Oxidative stress is a significant modulator in cancer progression and normal tissue injury. Advancements in monitoring redox status that identify potential targets of intervention may mitigate some off-target tissue injury seen in patients receiving various types of cancer treatment. Interest in EVs as an area of research has surged due to their various roles and functions in both normal and pathophysiological states. The current literature has provided insight into some of the roles they play and how they may facilitate disease, while also demonstrating their potential use in the clinic as a drug delivery tool and as a diagnostic marker. In particular, the study of the contents of EVs to monitor the status of the cell of origin, such as measuring oxidative modifications, may provide critical information to help facilitate better treatments for patients and help mitigate off-target consequences. We predict that utilization of EVs and redox status will provide a practical and early indicator of disease progression and treatment outcome, leading to the development of precision and targeted therapy. Further studies need to be conducted to optimize the use of EVs in the clinic and improve methods to promote rigor and reproducibility in studying EVs. We are optimistic that the importance of the clinical potential of redox status in EVs will ultimately be appreciated.

## Figures and Tables

**Table 1:** Characterization of EVs. The purpose of this table is to summarize the parameters outlined by MISEV2018. For optimal characterization of EVs, please refer to the original article by Thery et al. for detailed information about MISEV2018 guidelines [31]. The first column is the four different parameters that must be assessed when studying EVs; the second column gives the reasoning and purpose of assessing this parameter; the third column provides methods suggested by MISEV2018; and the fourth column cites any additional notes highlighted in the publication [31].

Parameter	Purpose	Suggested Methods	Additional Notes
1. Quantification of EVs	To best quantify the amount of EVs present in a sample. Starting volume that EVs are being isolated from also needs to be taken into account [31].	1. Protein concentration (e.g., BCA, Bradford, total protein on SDS-PAGE) 2. EVs’ particle number quantification (e.g., nanoparticle tracking analysis, standard or high-resolution flow cytometry)	Either total protein as measured by BCA or particle number is most commonly used. Quantification of total lipids, specific molecules, or total RNA may also be used.
2. General characteristics of EVs by protein composition	To quantify the purity of EVs isolation with a minimum of three positive markers (at least one transmembrane or lipid-bound protein and at least one cytosolic protein). Additionally, a negative or contamination marker must be used for a minimum total of at least four protein markers [31].	1. Western blotting 2. Flow cytometry 3. Capillary-based automated Western blot (Jess) [47] 4. Reverse phase protein array (RPPA) [48] 5. Mass spectrometry [49]	Two other categories of protein markers are suggested in MISEV2018 but are not required [31]: 1. Proteins localized in/on intracellular compartments of eukaryotic cells to identify specificity of small EVs’ subtype(s) (e.g., LMNA and CYC1) 2. Secreted or luminal proteins that can bind to receptors on the EV surface for mode of association of EVs (e.g., EGF, VEGFA, and collagen)
3. Characterization of single vesicles	Provide some parameters regarding the individual EVs present in the bulk population of EVs that are being used for study. Two methods must be used. The first should provide a high-resolution image of the EVs and the second should calculate biophysical parameters of single EVs that can be used to quantify a large number of EVs [31].	1. Electron microscopy (SEM, TEM, cryo-EM), SPM, Atomic Force Microscopy (AFM) [50], super-resolution microscopy 2. Nanoparticle tracking analysis, high-resolution flow cytometry, fluorescence correlation spectroscopy	The authors of MISEV2018 provide many potential methods that can be used for the characterization of single vesicles but emphasize the significance of proper documentation of the experimental conditions, such as documentation of the source of the EVs, the starting volume of the source, the conditions of isolation, etc.
4. Characterization of topology of EV-associated components	To determine the location of some proteins between the lumen and the surface of EVs [31].	1. Mild digestions, permeabilizations, or antibody studies followed by SDS-PAGE, RT-PCR, etc. 2. Flow cytometry and fluorescence microscopy with antibodies 3. EM with immunolabeling	Topology may be a result of unknown mechanisms localizing cytosolic components to the surface and may be important for function.

BCA, bicinchoninic acid; EM, electron microscopy; EVs, extracellular vesicles; MISEV, minimal information needed to study extracellular vesicles; SDS-PAGE, sodium dodecyl sulfate polyacrylamide gel electrophoresis; RT-PCR, reverse transcriptase polymerase chain reaction; SEM, scanning electron microscopy; SPM, scanning-probe microscopy; TEM, transmission electron microscopy.

**Table 2:** Methods of EVs isolation. Advantages and disadvantages of some methods currently used to isolate EVs are listed. This table reflects and aims to summarize the findings of Gardiner et al., Carnino et al., and Brennan et al. [53,54,55]. For original detailed information, please refer to their papers.

Method	Advantages	Disadvantages
Ultracentrifugation	Low cost Able to process high volume of sample No additional chemicals needed	Need access to ultracentrifugation equipment Tedious and time-consuming protocols Efficacy of isolation is dependent on rotor * Potential damage to EVs integrity
Density gradient centrifugation	Purity of isolation No additional chemicals needed	Need access to ultracentrifugation equipment Difficult to perform with small volume of initial material Loss of sample during isolation
Filtration	Straightforward protocol Can isolate from numerous samples at once No limitation on starting sample volume	Potential loss of sample Sample contamination Larger sample size may result in lower yield
Size exclusion chromatography	Purity of isolation Preservation of vesicle integrity Prevention of EV aggregates Able to isolate EVs based on size to differentiate between categories Short time for isolation	Only able to process small sample volumes Tedious protocols Need for specialized equipment

* Cvjetkovic et al. observed differences in EVs isolation purity when utilizing the same protocol for different rotor types, indicating that it is necessary to optimize protocols and calculate the purity and reproducibility of the isolated EVs [56].

## Abbreviations

AFM: atomic force microscopy; ALL: Acute Lymphoblastic Leukemia; CSF: cerebrospinal fluid; CNS: central nervous system; ESCRT: endosomal complexes required for transport machinery; EV: extracellular vesicles; GFAP: glial fibrillary acidic protein; GPx: glutathione peroxidase; HNE: 4-hydroxy-2-nonenal; IHC: immunohistochemistry; ILV: intraluminal vesicle; ISEV: International Society of Extracellular Vesicles; LPO: lipid peroxidation; MISEV: minimal information for studies of extracellular vesicles; MVB: multivesicular body; PYGB: glycogen phosphorylase; RNS: reactive nitrogen species; ROS: reactive oxygen species; SNARE: soluble NSF attachment protein receptor; SOD: superoxide dismutase.
